# Fatty acid profiles of five farmed Brazilian freshwater fish species from different families

**DOI:** 10.1371/journal.pone.0178898

**Published:** 2017-06-14

**Authors:** Bruna Leal Rodrigues, Anna Carolina Vilhena da Cruz Silva Canto, Marion Pereira da Costa, Flávio Alves da Silva, Eliane Teixeira Mársico, Carlos Adam Conte-Junior

**Affiliations:** 1 Departamento de Tecnologia de Alimentos, Faculdade de Veterinária, Universidade Federal Fluminense (UFF), Niterói, Rio de Janeiro, Brazil; 2 Programa de Ciência de Alimentos, Instituto de Química, Universidade Federal do Rio de Janeiro (UFRJ), Rio de Janeiro, Brazil; 3 Setor de Engenharia de Alimentos, Escola de Agronomia, Universidade Federal de Goiás (UFG), Goiás, Brazil; University of Illinois, UNITED STATES

## Abstract

The proximate composition and fatty acid (FA) profiles of five Brazilian freshwater fish species, namely *Brycon cephalus* (BC), *Cichla ocellaris* (CO), *Prochilodus lineatus* (PL), *Leporinus friderici* (LF) and *Pseudoplatystoma corruscans* (PCO), were investigated. CO and LF exhibited the highest (*p* < 0.05) moisture content, as well as one of the lowest (*p* < 0.05) lipid values, whereas BC presented the lowest (*p* < 0.05) moisture and, alongside PL, the highest (*p* < 0.05) lipid content. The predominant FAs in the evaluated fish species were palmitic, oleic, linoleic and docosahexaenoic acids. BC and CO presented high *n*-3 PUFA content, especially DHA, and demonstrated superior nutritional quality indices compared to the other evaluated fish species. Furthermore, a significant relationship was observed among these species, suggesting they possess similar nutritional lipid values. Thus, BC and CO were proven to be an excellent matrix with relevant lipid quality, desirable for human health.

## Introduction

Global fish supplies from aquaculture have increased steadily in recent years, with freshwater organisms, mostly from inland aquaculture activities, representing an important segment, of 41.9 million tones [1]. In South America, Brazil has shown significantly increases in this regard, and is considered one of the major aquaculture producers [2]. In 2010, Brazil contributed with 18.61% of the total aquaculture production and was considered one of 15 largest fish producers in 2012, contributing with 1.1% of the total farmed fish production in the world [1,2]. The potential of Brazilian aquaculture is due to its vast and varied natural resources, as well as great hydrographic basins and an enormous diversity of fish species with promising characteristics for meat production [3,4]. The potential for fish-farming, the growth of demanding consumers for a healthy diet in addition to the need to increment the current and future stocks of fish as a source of sustainable protein makes Brazilian native fish a potential alternative to contribute to the advance in freshwater aquaculture and to the worldwide increase consumption of Brazilian freshwater fish species [2,5].

Regarding Brazilian inland production, freshwater aquaculture activities are the most prominent, in which tilapia, carp and some Brazilian freshwater species from the Serrasalmidae family accounted for 87% of the total aquaculture production in 2011 [3,6]. Several other important Brazilian fish families, such as Bryconidae, Pimelodidae, Cichlidae, Prochilodontidae and Anostomidae, also exhibit great potential regarding fish farming and are highly accepted by North and Midwest consumers [7–11]. However, Brazilian freshwater fish production is still under-exploited, offering low amounts and a low variety of fish to Brazilian and worldwide populations [8]. Brazilian and Western world fish consumption are still modest when compared to other meat matrices and its diets are characterized by low consumption of foods containing high amounts of *n*-3 fatty acids and a high intake of foods rich in saturated (SFA) and *n*-6 fatty acids. Low fish consumption increases dietary imbalances, which are closely associated to damaging effects on human health, mainly to the cardiovascular system [12]. In 2011, more than one hundred thousand deaths were caused by circulatory system diseases, considered one of the main mortality causes in Brazil [13,14].

Fish are an important component of human nutrition and the benefit of this meat matrix is highly associated to the quality of their lipid content, a source of essential polyunsaturated fatty acids (PUFA), mainly eicosapentaenoic acid (EPA- C20:5 n3), docosahexaenoic acid (DHA- C22:6 n3) and linoleic acid (C18:2 n6) [15,16]. *n*-3 PUFA’s, mainly EPA and DHA, have been widely reported to promote several benefits on human health, especially regarding the prevention of cardiovascular disorders [15]. Moreover, recent studies have also suggested that the *n*-6 family and its metabolites show beneficial effects on cardiovascular system health [16,17], contradicting the massive association between this FA and the progress of this type of disease [12]. Nevertheless, it has been established that a well-balanced diet of *n*-3/*n*-6 FAs (1/1 to 1/2) is highly recommended [18]. In addition, allied to *n*-3/*n*-6 PUFA ratios, several other indices have been developed in order to provide further knowledge on fish lipid quality and its association with cardiovascular disorders [19,20,21]. Thus, the increase of fish consumption can improve the quality of western diets, decreasing the risk of several disorders [19,20,22].

Previous studies have established that specific habits and characteristics from different fish families, such as type of diet, nocturnal/diurnal habits and deep/shallow water preferences lead to significant differences in fish anatomy and physiology [23,24]. Moreover, herbivorous, omnivorous and carnivorous species generally present differences in intestine length and morphology, which influence nutrient digestion and absorption processes [25,26,27]. In addition, species belonging to lower trophic levels, such as *Prochilodus lineatus* (Curimbatá), *Brycon cephalus* (Matrinxã) and *Leporinus friderici* (Piau), digest their diets more easily when compared to carnivorous fish like *Cichla ocellaris* (Tucunaré) and *Pseudoplatystoma corruscans* (Pintado) [28]. These distinct characteristics influence fish metabolic apparatuses and, consequently, the fatty acid composition of each species [25,28]. However, scientific reports comparing fatty acid profiles of different Brazilian freshwater fish families are still scarce. Therefore, in order to compare species from different trophic guild, indicate those with better nutritional quality for human consumption and encourage more research in this regard to enhance the national production and worldwide consumption of Brazilian fish species, the present study aimed to evaluate the proximate composition and fatty acid profiles of fish families with high acceptance in the North and Midwest regions of Brazil.

## Materials and methods

### Sample collection and experimental design

Five fish species belonging to different freshwater families with distinct characteristics and feeding habits, namely deep water detritivorous fish with diurnal habits (Prochilodontidae family: *Prochilodus lineatus* (Curimbatá)–PL) [29], shallow water omnivorous fish with diurnal habits (Bryconidae family: *Brycon cephalus* (Matrinxã)–BC and Anostomidae family: *Leporinus friderici* (Piau)–LF) [9,30], shallow water carnivorous fish with diurnal habits (Cichlidae family: *Cichla ocellaris* (Tucunaré)–CO) [31] and deep water carnivorous fish with nocturnal habits (Pimelodidae family: *Pseudoplatystoma corruscans* (Pintado)–PCO) [10], were obtained from Turvale fish farms in Firminópolis, Goiás, Brazil (16° 34′ 55″ S, 50° 18′ 18″ W). Although they present different characteristics and habits, the fish were all fed the same diet (Criapeixe E200, Guabi, SP, Brazil) and farmed in the same conditions, in order to verify the potential conversion of feed into fatty acids in muscle tissue. However, in addition to a diet composed of artificial food, the carnivorous fish species were cultivated alongside low commercial value species with the purpose of maintaining baseline physiological needs. This study was carried out in strict accordance with the recommendations of the Guide for the Care and Use of Laboratory Animals of the National Institutes of Health (USA) and the Committee on the Ethics of Animal Experiments of the Federal Fluminense University, Rio de Janeiro, Brazil (2016.12.05CEUA). The fish were sacrificed by thermal shock through ice slurry immersion [22, 32] and all efforts were made to minimize suffering. The samples were then transported to the laboratory on ice (0°C) in styrofoam boxes, where a total of 20 fish were weighed and measured. Each individual fish was immediately beheaded and gutted and the muscle tissue was then filleted. The samples were kept frozen (- 80°C) until the tests were performed, in order to prevent fatty acid oxidation. The proximate composition and fatty acid profiling of the fish fillets were obtained from four trials (n = 4), totaling 8 experimental replicates for each species.

### Proximate composition

The proximate composition of the fish fillets was determined according to AOAC [33], where moisture content was assessed by drying the samples in an oven at 100–105°C, protein content was determined by the Kjeldahl method and ash content was estimated after incineration at 550°C in a muffle furnace. Total lipids were analyzed using the Bligh and Dyer method [34], with slight modifications. Briefly, lipids were extracted from 5 g samples by methanol:chloroform (2:1 vol/vol) followed by three successive washes with chloroform, in order to recover maximum lipid content. The extracts were then centrifuged at 3,000 × *g* for 15 min (Hermle Z 360K, Wehingen, Germany), evaporated and gravimetric determinations were then conducted. The results were expressed as percentage of total lipids (%).

### Fatty acid profiles

The total lipids of fish samples were extracted based on the method reported by Bligh & Dyer [34] with slight modifications, as described previously. In brief, lipids were extracted from samples (5 g) by methanol:chloroform (2:1 vol/vol) and followed by three successive washes with chloroform, in order to recover maximum lipid content. The extracts were centrifuged at 3,000 × *g* for 15 min (Hermle Z 360K, Wehingen, Germany) and evaporated in nitrogen flow. Methylation was conducted in an acidic media (HCl 10% in methanol) in a water bath at 60°C (Novatecnica, NT247, São Paulo, Brazil). Furthermore, 1 mL of hexane was added, the supernatant was removed and then conditioned in vial [35,36]. The derivatized fatty acid methyl esters (FAMEs) were determined on a gas chromatograph equipped with a flame ionization detector (Perkin Elmer, Waltham, MA, USA) and an Omegawax 320 column (30 m length, 0.32 mm internal diameter, and 0.25 μm particle size) (Supelco Inc., Bellefonte, PA, USA). Two microliters of sample and a 1:20 split were used. The injector and detector temperatures were set at 260°C and 280°C, respectively. The initial temperature of the oven was programmed to 110°C, increasing by 40°C/min until 233°C with 2 min holding time, after which the temperature was further increased to 240°C by 1°C/min and held for 21 min. Helium was used as the carrier gas at a flow rate of 1.8 mL/min (10 psi). FAMEs were identified comparing their retention time with the commercial standard mixtures FAME Mix, C4-C24 analytical standard (Supelco Inc., Bellefonte, PA, USA). The fatty acid profile was determined by an internal normalization determination. The response factors of all fatty acids were calculated and the corrected peak areas were used to determinate a relative percentage of total area from identified FAMEs, following the equation: % FAX = [AX/AR], where FAX = quantified fatty acid; AX = area of X and AR methyl esters = total area of the chromatogram [19].

### Nutritional quality indices

The data from the fatty acid compositions were used to investigate the nutritional lipid quality indices from the five evaluated freshwater fish species through three important indicators, namely, index of atherogenicity (IA) and index of thrombogenicity (IT), according to Ulbricht and Southgate [37] with modifications, and the hypocholesterolemic/hypercholesterolemic ratio (h/H) according to Santos-Silva, Bessa, and Santos-Silva [38], as follows:

$$\begin{array}{l} IA=\left(4\times C14:0+C16:0\right)/\left(\sum MUFA+\sum \left(n–6\right)+\sum \left(n–3\right)\right)\\ IT=\left(C14:0+C16:0+C18:0\right)/\left(0.5\times \sum MUFA+0.5\times \sum \left(n–6\right)+3\times \sum \left(n–3\right)+100\times \sum \left(n–3\right)/\sum \left(n–6\right)\right)\\ h/H=\left(C18:1n9+C18:2n6+C20:4n6+C18:3n3+C20:5n3+C22:5n3+C22:6n3\right)/\left(C14:0+16:0\right) \end{array}$$

### Statistical analyses

The morphometric parameter, proximate composition and fatty acid profile data of the five Brazilian freshwater fish were subjected to a one-way ANOVA. Tukey’s test was used to determine differences between means at a 0.05 level of significance. A Principal Component Analysis (PCA) was conducted on the specific FA, total FAs and nutritional quality indices to identify clustering. Pearson´s correlation test was selected to perform correlations analysis. All analyses were performed using the XLSTAT software package (Version 2012.6.08, Addinsoft, Paris, France).

## Results

### Proximate composition

Table 1 displays the morphometric parameters and proximal composition of the five species evaluated herein. Differences were observed for length and weight; in which PCO presented the greatest values (*p* < 0.05) and LF, the lowest (*p* < 0.05).

**Table 1:** Morphometric data and proximate composition of the five Brazilian freshwater fish species evaluated herein.

Parameters	Freshwater fish species*				
	BC	CO	PL	LF	PCO
**Morphometric**					
Length (cm)	30.50±1.08^b^	36.17±0.89^c^	35.17±1.26^bc^	25.07±1.81^a^	56.50±4.15^d^
Weight (g)	915.47±40.15^c^	874.62±37.94^c^	559.10±44.85^b^	200.54±43.60^a^	1199.64±290.49^d^
**Proximate composition** (%)					
Moisture	72.61±0.64^a^	78.27±0.25^d^	75.59±0.23^c^	77.98±0.15^d^	74.46±0.40^b^
Protein	23.48±0.61^b^	24.50±0.84^b^	20.76±0.79^a^	19.72±0.53^a^	23.51±0.52^b^
Lipids	2.72±0.36^c^	1.32±0.07^b^	2.38±0.24^c^	1.17±0.09^b^	0.54±0.18^a^
Ash	1.07±0.03^a^	1.10±0.06^a^	1.19±0.07^ab^	1.30±0.06^bc^	1.40±0.06^c^

* Freshwater fish species: *Brycon cephalus*–BC, *Cichla ocellaris–*CO, *Prochilodus lineatus*–PL, *Leporinus friderici*–LF, *Pseudoplatystoma corruscans*–PCO

Results are expressed as least square means ± standard deviation.

Different lowercase letters (a–d) indicate significant differences between fish species (*p* < 0.05)

The moisture content of all five species ranged from 72.61% in BC to 78.27% in CO. The highest (*p* < 0.05) fat content was found in BC and PL whereas the lowest (*p* < 0.05) was verified in PCO. Protein values ranged from 19.72% in LF to 24.50% in CO, wherein BC, CO and PCO presented the highest (*p* < 0.05) protein results. Regarding ash content, the values varied from 1.07% to 1.40%.

### Fatty acid profiles

The fatty acid composition of the fillets from the five Brazilian freshwater fish species evaluated herein are displayed in Table 2. BC, CO and LF exhibited similar patterns (PUFA > MUFA > SFA), whereas PL presented the same MUFA and SFA content and PCO had higher SFA content in relation to MUFA values.

**Table 2:** Fatty acid profile (% of total fatty acids) and nutritional quality indices in fillets of five Brazilian freshwater fish species.

Fatty Acids	Freshwater species*				
	BC	CO	PL	LF	PCO
C14:0	0.48±0.14^b^	0.36±0.02^ab^	0.81±0.08^c^	0.26±0.03^a^	0.45±0.12^ab^
C16:0	13.01±1.29^a^	16.37±0.81^b^	22.35±0.70^d^	18.77±0.76^c^	18.32±0.96^bc^
C18:0	0.81±0.25^b^	0.73±0.06^ab^	0.49±0.01^a^	0.86±0.04^b^	0.72±0.02^ab^
C20:0	ND	ND	ND	ND	ND
C22:0	1.99±0.27^b^	ND	0.34±0.07^a^	1.11±0.10^ab^	4.87±1.12^c^
C24:0	0.71±0.36^a^	ND	ND	ND	0.43±0.05^a^
C16:1	4.29±0.83^a^	4.76±0.24^ab^	6.50±0.20^c^	5.46±0.22^b^	5.33±0.28^b^
C18:1n7	4.37±1.04^b^	3.91±0.32^b^	2.61±0.05^a^	4.59±0.20^b^	3.86±0.08^b^
C18:1n9	12.66±1.58^bc^	12.28±1.01^b^	8.19±0.16^a^	14.41±0.62^c^	12.11±0.25^b^
C22:1n9	0.73±0.55^a^	2.07±0.21^b^	1.31±0.45^a^	1.03±0.04^a^	ND
C24:1	2.24±1.32^a^	3.09±1.33^a^	3.83±1.05^a^	2.60±0.36^a^	1.68±0.36^a^
C18:2n6	19.90±0.99^b^	14.09±0.81^a^	12.97±0.47^a^	26.24±0.17^d^	23.91±1.61^c^
C18:3n6	5.77±1.66^b^	6.74±1.74^b^	7.18±0.42^b^	2.06±0.02^a^	0.45±0.21^a^
C18:3n3	0.71±0.31^a^	0.21±0.13^a^	0.75±0.12^a^	0.25±0.03^a^	0.85±0.66^a^
C20:2	2.60±0.55^b^	1.36±1.13^ab^	0.33±0.65^a^	ND	2.10±0.41^b^
C20:3n6	ND	ND	ND	ND	ND
C20:3n3	0.30±0.21^a^	ND	ND	ND	ND
C20:4n6	0.71±0.44^a^	1.01±0.89^a^	6.23±0.38^b^	0.33±0.02^a^	0.72±0.54^a^
C20:5n3	6.19±1.20^a^	7.34±0.95^a^	7.44±0.39^a^	6.41±0.10^a^	6.12±0.18^a^
C22:2	0.78±0.37^b^	0.27±0.32^a^	ND	ND	ND
C22:6n3	21.73±1.70^c^	25.42±0.85^d^	18.65±0.99^b^	15.63±0.51^a^	18.08±0.75^b^
**Ʃ SFA**	17.01±1.39^aA^	17.45±0.83^aA^	23.99±0.56^cA^	21.00±0.62^bA^	24.80±0.39^cB^
**Ʃ MUFA**	24.29±0.69^abB^	26.11±0.75^bcB^	22.45±1.51^aA^	28.09±0.97^cB^	22.97±0.90^aA^
**Ʃ PUFA**	58.70±1.41^cC^	56.44±1.15^cC^	53.56±1.17^bB^	50.91±0.73^aC^	52.23±0.64^abC^
**Ʃ n-3**	28.94±1.13^c^	32.97±0.80^d^	26.85±1.06^bc^	22.29±0.58^a^	25.05±1.50^b^
**Ʃ n-6**	26.38±1.04^bc^	21.83±1.62^a^	26.38±0.76^bc^	28.63±0.16^c^	25.08±1.28^b^
**PUFA/SFA**	3.47±0.38^b^	3.24±0.21^b^	2.23±0.05^a^	2.43±0.08^a^	2.11±0.03^a^
**n-3/n-6**	1.10±0.06^b^	1.52±0.12^c^	1.02±0.07^b^	0.78±0.02^a^	1.00±0.11^b^
**n-6/n-3**	0.91±0.05^b^	0.66±0.05^a^	0.98±0.06^b^	1.28±0.03^c^	1.01±0.11^b^
**EPA+DHA**	27.93±1.28^c^	32.76±0.87^d^	26.10±1.08^bc^	22.04±0.57^a^	24.20±0.85^b^
**DHA/EPA**	3.63±0.89^c^	3.51±0.53^bc^	2.51±0.19^ab^	2.44±0.07^a^	2.95±0.11^abc^
**IA**	0.19±0.00^a^	0.22±0.00^b^	0.34±0.00^e^	0.25±0.00^c^	0.28±0.00^d^
**IT**	0.06±0.01^a^	0.06±0.00^a^	0.11±0.01^c^	0.11±0.00^c^	0.10±0.01^b^
**h/H**	4.62±0.47^c^	3.61±0.17^b^	2.34±0.06^a^	3.33±0.15^b^	3.30±0.16^b^

* Freshwater fish species: *Brycon cephalus*–BC, *Cichla ocellaris–*CO, *Prochilodus lineatus*–PL, *Leporinus friderici*–LF, *Pseudoplatystoma corruscans*–PCO

ND = Not detected

SFA = saturated fatty acid; MUFA = monounsaturated fatty acid; PUFA = polyunsatutaed fatty acid; EPA = eicosapentaenoic acid; DHA = docosahexaenoic acid; IA = Index of Atherogenicity; IT = Index of Thrombogenicity; h/H = Hypocholesterolemic/hypercholesterolemic ratio

Results are expressed as least square means ± standard deviation

Different lowercase letters (a–d) indicate significant differences among fish species (*p* < 0.05)

Different uppercase letters (A–C) indicate significant differences among the total fatty acids (*p* < 0.05)

The highest (*p* < 0.05) total SFA values were observed in PL and PCO, whereas the lowest (*p* < 0.05) were observed in BC and CO. Palmitic acid (C16:0) was the most abundant SFA in all studied species, representing more than 70% of total SFAs. Myristic acid (C14:0), on the other hand, was observed in low concentrations in all species. In relation to MUFA, the lowest (*p* < 0.05) values were observed in PL, which exhibited one of the greatest (*p* < 0.05) lipid content among the investigated species. Oleic acid (C18:1 n9) was the predominant fatty acid in all species, ranging from 36.47% (PL) to 52.72% (PCO) of total MUFAs. All fish species demonstrated high PUFA content, ranging from 50.91% in LF to 58.70% in BC. The *n*- 3 FA represented a large proportion (43.78–58.41%) of total PUFA values. DHA accounted for 30.70 to 45.04% whereas EPA represented 10.54–13.89% of total PUFA contents. Among the evaluated freshwater fish, CO presented the lowest (*p* < 0.05) total *n*-6 values and, with PL the lowest (*p* < 0.05) linoleic acid—LA (C18:2 n6) values, corroborating previous findings. Total *n*-6 FA accounted from 38.67–56.24% of total PUFA, in which LA was the predominant FA, representing 49.17–95.33% of total *n*-6 PUFAs. Regarding arachidonic acid (C20: 4n-6), PL demonstrated the greatest (*p* < 0.05) values, whereas BC, CO, LF and PCO showed the lowest (*p* < 0.05).

### Nutritional quality indices

The nutritional quality indices (NQI) of the five freshwater Brazilian fish species are displayed in Table 2. PUFA/SFA ratio ranged from 2.11 in PCO to 3.47 in BC, whereas the *n*-6/*n*-3 ratio varied from 0.66 in CO to 1.28 in LF. Regarding the *n*-3/*n*-6 ratio, CO (1.52) presented the highest (*p* < 0.05) value. In the present study, BC (0.19) presented the lowest (*p* < 0.05) IA value, while BC (0.06) and CO (0.06) presented the lowest (*p* < 0.05) IT values. Among the evaluated species, BC (4.62) presented the greatest (*p* < 0.05) h/H values.

### Principal component analysis

A Principal Component Analysis (PCA) was performed on the specific FA, total FAs (SFA, MUFA, PUFA, EPA+DHA, *n*-3 and *n*-6 PUFA) and NQI (IT, IA, h/H, *n*-6/*n*-3, *n*-3/*n*-6, PUFA/SFA and DHA/EPA) data. Pearson correlations among the specific FA, total FA and the NQI of five Brazilian freshwater fish species demonstrated a strong correlation among several of the evaluated parameters (Table 3). The main positive correlation (*p* < 0.05) was observed between PUFA and DHA/EPA (r = 0.89) as well as between *n*-3/*n*-6 and EPA+DHA (r = 0.98), whereas the main negative correlation (*p* < 0.05) was verified between IT and PUFA (r = - 0.89); IT and PUFA/SFA (r = - 0.90); h/H and IA (r = -0.96) as well as IT and DHA/EPA (r = - 0.99).

**Table 3:** Results of Pearson’s correlation test among the specific FA, total FAs and NQI of five Brazilian freshwater fish species.

	SFA	MUFA	PUFA	*n*-3	*n*-6	PUFA/SFA	*n*-3/n-6	*n*-6/n-3	EPA+DHA	DHA/EPA	IA	IT	h/H
C14:0	0.44	-0.83	0.11	0.09	0.04	-0.28	-0.03	-0.14	0.04	-0.23	0.68	0.27	-0.53
C16:0	0.81	-0.25	-0.73	-0.43	0.23	-0.85	-0.36	0.36	-0.41	-0.87	**0.97**	0.85	**-0.99**
C18:0	-0.54	0.72	0.08	-0.16	0.19	0.42	-0.14	0.25	-0.14	0.32	-0.81	-0.34	0.75
C20:0													** **
C22:0	0.46	-0.38	-0.24	-0.40	0.11	-0.37	-0.34	0.24	-0.45	0.04	-0.02	0.08	0.18
C24:0	-0.22	-0.37	0.52	0.05	0.08	0.37	-0.07	-0.09	-0.02	0.58	-0.51	-0.47	0.73
C16:1	0.80	-0.33	-0.67	-0.46	0.32	-0.82	-0.42	0.39	-0.45	-0.86	**0.98**	0.86	**-0.97**
C18:1n7	-0.54	0.72	0.08	-0.16	0.19	0.42	-0.14	0.24	-0.14	0.32	-0.81	-0.34	0.76
C18:1n9	-0.45	0.79	-0.07	-0.22	0.16	0.30	-0.15	0.29	-0.18	0.21	-0.73	-0.24	0.64
C22:1n9	-0.51	0.39	0.29	0.62	-0.45	0.43	0.62	-0.49	0.67	0.15	-0.08	-0.27	-0.14
C24:1	0.00	-0.05	0.04	0.28	-0.09	-0.02	0.23	-0.20	0.31	-0.30	0.49	0.22	-0.58
C18:2n6	0.18	0.41	-0.50	-0.75	0.58	-0.28	-0.69	0.73	-0.74	-0.26	-0.24	0.30	0.28
C18:3n6	-0.47	-0.17	0.66	0.72	-0.35	0.54	0.58	-0.61	0.71	0.33	-0.02	-0.39	-0.02
C18:3n3	0.53	**-0.91**	0.07	-0.18	0.16	-0.33	-0.27	0.05	-0.27	-0.02	0.36	0.15	-0.10
C20:2	-0.33	-0.39	0.66	0.42	-0.39	0.50	0.36	-0.49	0.36	0.82	-0.58	-0.74	0.73
C20:3n6													** **
C20:3n3	-0.60	-0.12	0.76	0.24	0.16	0.70	0.03	-0.14	0.18	0.63	-0.65	-0.58	0.81
C20:4n6	0.46	-0.59	-0.08	0.04	0.07	-0.37	-0.04	-0.06	0.02	-0.43	0.80	0.44	-0.74
C20:5n3	-0.02	-0.09	0.09	0.50	-0.44	0.02	0.51	-0.47	0.53	-0.12	0.43	0.04	-0.57
C22:2	-0.80	-0.01	**0.92**	0.53	-0.14	**0.90**	0.36	-0.43	0.49	0.83	-0.79	-0.81	0.87
C22:6n3	-0.69	-0.04	0.82	**0.99**	-0.86	0.77	**0.97**	**-0.96**	**0.99**	0.85	-0.51	**-0.89**	0.42
SFA	**1.00**	-0.50	-0.77	-0.63	0.30	**-0.97**	-0.54	0.47	-0.64	-0.75	0.87	0.82	-0.78
MUFA		**1.00**	-0.17	-0.13	0.16	0.28	-0.06	0.27	-0.05	-0.05	-0.48	-0.05	0.28
PUFA			**1.00**	0.81	-0.46	**0.90**	0.65	-0.73	0.77	**0.89**	-0.63	**-0.89**	0.68
*n*-3				**1.00**	-0.86	0.72	**0.97**	**-0.97**	**1.00**	0.80	-0.40	-0.83	0.33
*n*-6					**1.00**	-0.38	**-0.95**	**0.93**	-0.86	-0.64	0.21	0.66	-0.11
PUFA/SFA						**1.00**	0.61	-0.59	0.72	0.86	-0.85	**-0.90**	0.81
*n*-3/*n*-6							**1.00**	**-0.97**	**0.98**	0.74	-0.35	-0.78	0.24
*n-6/n-3*								**1.00**	**-0.96**	-0.77	0.30	0.79	-0.24
EPA+DHA									**1.00**	0.77	-0.40	-0.82	0.30
DHA/EPA										**1.00**	-0.79	**-0.99**	0.80
IA											**1.00**	0.81	**-0.96**
IT												**1.00**	-0.78
h/H													**1.00**

SFA = saturated fatty acid; MUFA = monounsaturated fatty acid; PUFA = polyunsatutaed fatty acid; EPA = eicosapentaenoic acid; DHA = docosahexaenoic acid; IA = Index of Atherogenicity; IT = Index of Thrombogenicity; h/H = Hypocholesterolemic/hypercholesterolemic ratio.

**Fig 1:**
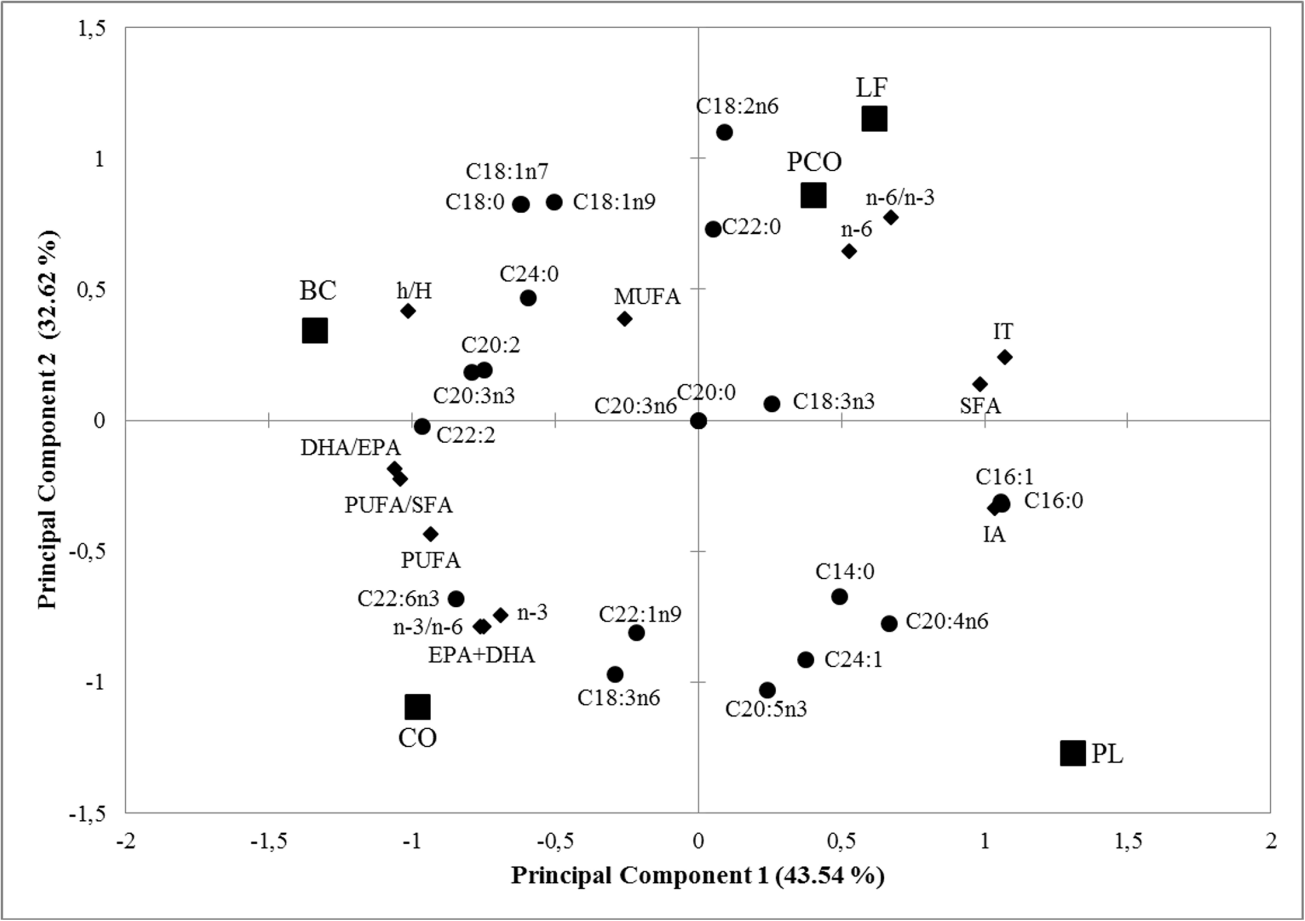
Specific FA, total FAs and NQI of the different freshwater fish species in the plane defined by two principal components BC (*Brycon cephalus*); CO (*Cichla ocellaris*); PL (*Prochilodus lineatus*); LF (*Leporinus friderici*); PCO (*Pseudoplatystoma corruscans*). SFA (Saturated fatty acid); MUFA (Monounsaturated fatty acid); PUFA (Polyunsatutaed fatty acid); EPA (Eicosapentaenoic acid); DHA (Docosahexaenoic acid); IA (Index of Atherogenicity); IT (Index of Thrombogenicity); h/H (Hypocholesterolemic/hypercholesterolemic ratio).

The PCA explained 76.16% of the observed variance (Fig 1). The principal component-1 (PC1) was predominant and contributed to 43.54% of the explained variance, whereas the principal component-2 (PC2) contributed to 32.62%. In general, PC1 separated and clustered the species into two groups (BC and CO; LF, PCO and PL) and Pearson correlations confirmed that these species were clearly divided based on PUFA, MUFA, *n*-3 PUFA and their associated NQI (h/H, *n*-3/*n*-6, PUFA/SFA, EPA+DHA and DHA/EPA) and on SFA, *n*-6 PUFA and their associated NQI (IT, IA and *n*-6/*n*-3), respectively.

## Discussion

Few studies on the lipid portion comparison of Brazilian freshwater fish species from different trophic guilds are available, as well as those that discuss the lipid and nutritional quality for human consumption, leading to the low knowledge and consumption of these fish species worldwide. Currently, Brazil is among the major aquaculture producers in South America, and one of the 15 largest global fish producers, due to the potential of Brazilian fish aquaculture [1]. However, although the Brazilian fish farming be promising due to its extensive natural resources and variety of fish species with favorable zootechnical characteristics, native fish species are until underexplored [3,4]. The exploration of these resources converges with the growth of demanding consumers for healthy diets with high amounts of PUFA, from both sustainable and natural sources, which preserves distinct characteristics and flavor [5]. These facts make Brazilian native fish a potential alternative to attend the demanding population and effectively contribute to the advance of Brazilian aquaculture and increase of worldwide fish consumption. Overall, the major finding of the present study was that BC and CO presented high *n*-3 PUFA content, as well as superior NQI, among the evaluated fish species. In addition, a significant relationship was verified between these species, suggesting similar nutritional lipid quality. Nevertheless, the NQI of all the evaluated species were within the recommended standard, suggesting that their consumption can positively contribute to human health.

Regarding the proximate composition of the five Brazilian freshwater fish species evaluated herein, similar moisture results were found by Rodrigues et al. [39] in farmed rainbow trout (*Oncorhynchus mykiss*) as well as by Memon et al. [19] and Jabeen and Chaudhry [22] in three farmed carp species (*Labeo rohita*, *Cirrhinus mrigala*, *Catla catla*) and in wild carp and tilapia (*Cyprinus carpio*, *Labeo rohita*, *Oreochromis mossambicus*), respectively. The data reported for Brazilian freshwater fish species, such as *Brycon cephalus* cultured in ponds (73.02%) and cages (71.77%) [7] and *Cichla* sp. sampled during different seasonal periods (76–78%) [11], also corroborate with the results presented herein. Concerning lipid content, PL is a detritivorous fish reported to present specific anatomical and histological intestinal characteristics, such as numerous absorptive pyloric caeca and a long intestine, which could promote more intensive nutrient absorption [29]. In addition, the Brycon (BC) genus presents a short adaptation time of the intestinal enzyme secretion metabolism (amylase, trypsin and lipase) which adjusts the amount of enzymatic apparatus to protein, fat and carbohydrate content, allowing its efficient use and probable effective absorption of these compounds when feeding [40]. Moreover, both species (PL and BC) exhibit a long, coiled and slim intestine, which favors absorption when compared to carnivorous fish (PCO), that possess short, straight and thick intestines [26,41], which explain the highest (*p* < 0.05) fat content in BC and PL and the lowest (*p* < 0.05) one in PCO. Nevertheless, according to Osman, Suriah, and Law [42], some fish species exhibit a reverse relationship between fat and moisture content, in which low fat fishes present greater water values, which was verified herein for BC (Table 1) and also confirmed by Memon et al. [19] for *C*. *catla* and *L*. *rohita* and by Jabeen and Chaudhry [22] for *L*. *rohita* and *O*.*mossambicus*. Regarding lipid content classification [43], CO, LF and PCO were considered very low (<2% fat) and BC and PL low (2–4% fat) fat fishes. Similar lipid content results were observed in different Brazilian freshwater fish species by Andrade et al. [44] for *Prochilodus lineatus* (3.68%), *Leporinus friderici* (2.60%) and *Pseudoplatystoma corruscans* (1.27%) purchased from fish markets in the state of Paraná and Inhamuns et al. [11] for Cichla sp. (0.8–2.1%) sampled during different seasons in the Amazon Basin. Unlike the species cited above, the lipid content data of BC (2.72%) was conflicting when compared to previous studies. Moreira et al. [7] found values ranging from 5.09 to 6.83% for *B*. *cephalus* cultured in ponds and cages, whereas Almeida et al. [45] reported 0.94% for *B*. *cephalus* farmed in the Amazon area. These variations are probably related to different farming conditions and the type of diet provided [45].

Although carnivorous fish (CO and PCO) possess short, straight and thick intestines and ingest lower amounts of food, the quality of the ingested feed is higher [26]. In addition, carnivores possess higher amount and activities of peptidases and proteases as well as numerous deep folds in the intestinal mucosa, which also increase mainly the peptide and amino acid absorption in this species, leading to variations in the final content of protein in fish fillets [26,41,46,47]. However, all investigated species reached values higher than 15% protein content, thus being classified as high protein foods [48]. The protein values of the present study are similar to the results obtained in previous studies on different freshwater fishes species, such as *B*. *cephalus* cultured in ponds (18.84%) and cages (20.03%) [7], *O*. *mykiss* (18.28%) [39] and *L*. *rohita*, *C*. *mrigala*, *C*. *catla* (20–23.57%) [19]. In relation to ash content, the values corroborates with those reported by previous studies for *B*. *cephalus* cultured in ponds (1.17%) and cages (1.15%) [7], *O*. *mykiss* (1.16%) [39] and *L*. *rohita*, *C*. *mrigala*, *C*. *catla* (1.12–1.21%) [19]. In general, the proximal composition variations verified between studies are considered common due to changes in fish age, sex, environment, geographical origin, season and diet [49,50].

Regarding SFA values, palmitic acid (C16:0) was the most abundant SFA in all studied species. The presence and content of these FA have been usually associated to planktonic or bacterial sources and they are commonly present in fish fat [51]. Myristic acid (C14:0) has been implicated in hypercholesterolemia in humans [20], although low amounts are beneficial to human health. In relation to MUFA values, oleic acid (C18:1 n9) was the predominant fatty acid in all species. However, the lowest (*p* < 0.05) values were observed in PL, which exhibited one of the greatest (*p* < 0.05) lipid content among the investigated species. These values are similar to those found by Fernandes et al. [20], that observed lower oleic acid values in sardines, a species with greater fat content. Oleic acid in fish is directly related to dietary FA content [43] and depends on the metabolism of each species [52], which may lead to variations in the final FA amount in fish fillets. High palmitic acid (C16:0) and oleic acid (C18:1 n9) contents have also been observed in previous studies conducted on freshwater fishes species, such as *L*. *rohita*, *C*. *mrigala*, *C*. *catla* [19] and *O*. *mykiss* [21,53], as well as *Cichla* sp.[11], *Prochilodus* spp.[8] and *P*. *corruscans* [44].

All fish species demonstrated high PUFA content, corroborating with previous studies conducted on freshwater fish species, such as *O*. *mykiss* [21,53], *L*. *rohita*, *C*. *mrigala*, *C*. *catla* [19] and *B*. *cephalus* [7]. The *n*- 3 FA represented a large proportion of total PUFA values, wherein DHA and EPA accounted for 30.70 to 45.04% and 10.54–13.89% of total PUFA contents, respectively. Dietary intake of DHA and EPA plays an important role in human health, reported as preventing several diseases, mainly of the cardiovascular system [12,29]. Therefore, high values of these PUFA in human diets are desirable. These high DHA and EPA values are similar to those observed by Özogul, Özogul and Alagoz [54] in *Clarias gariepinus*, *Cyprinus carpio*, *Siluris glanis*, *Tinca tinca*, *Sander lucioperca*; Memon et al. [19] in *L*. *rohita*, *C*. *mrigala*, *C*.*catla* as well as by Andrade et al. [44] in *P*. *lineatus*, *L*. *friderici*, *P*. *corruscans* and Inhamuns et al. [11] in *Cichla* sp.. Among the evaluated fish species, CO presented the highest (*p* < 0.05) total *n*-3 and EPA+DHA content, in addition to the highest (*p* < 0.05) DHA proportion. According to Inhamuns et al. [55], the high DHA and EPA+DHA content in *Cichla* sp. fillets is due to the fact that this species presents high amounts of D4-desaturase, which promotes an intensive conversion of EPA–DHA and maintains specific metabolic functions that result in DHA accumulation in fish fillets. Moreover, freshwater fish species have the ability to convert C18:2 n6 and C18:3 n3 into C:20 and C:22 equivalents (eicosanoid forms) through subsequent desaturation and chain elongation, as well as through enzymatic activity, resulting in high EPA and DHA contents and lower *n*-6 and linoleic acid (LA) values [19]. Linoleic acid—LA (C18:2 n6) and arachidonic acid (C20: 4 n6) are derived from farmed fish diets [19,56] that, associated with the metabolism of each species [52], influence the final FA content of fish fillets. LA and arachidonic acid are precursors of longer-chain *n*-6 PUFA [57] and these FA derivatives are frequently involved in cardiovascular disorders, such as thrombus and atheroma formation, leading to decreased human health [12,17]. Thus, low contents of these compounds are desired. In the present study high values of these FA was observed, corroborant with previous reports that have also found high LA and low arachidonic acid content in freshwater fish species, including *B*. *cephalus*, *B*. *microlepis*, *B*. *orbignyanus* [7] and *L*. *rohita*, *C*. *mrigala* and *C*. *catla* [19].

It is well established that the amount of some relevant FAs in fish fillets is strongly associated to their dietary source, especially in farmed fish [22,52,56], explaining the high content of, mainly, EPA, palmitic, oleic and linoleic acid in all analyzed species. However, freshwater and marine fishes can also synthesize MUFA and PUFA, as well as EPA and DHA, through enzymatic pathways characterized by sequential desaturation and elongation actions [58]. Thus, in addition to fish diet, genetic potential and fatty acid enzymatic biosynthesis, can also influence the final FA profile in muscle tissue [52,58], which can vary between species. Marine species present low enzymatic activity and depend almost completely on their diet to obtain the main long-chain *n*-3 FAs (EPA and DHA). In contrast, some freshwater species present unique enzymes with two desaturase activities, as well as multiple desaturase genes, which allow for PUFA biosynthesis [58]. Therefore, despite obtaining some FA from feed ingredients, these fish species also obtain these compounds through endogenous biosynthesis [52], explaining the differences among freshwater fish species, as well as the higher PUFA amounts, mainly EPA and DHA, in all analyzed species.

NQI have been developed to allow for the evaluation and comparison of fish lipid nutritional values, as well as for estimations of their effects on human health [19,20,53,54,59]. Among these indices, *n*-3/*n*-6, *n*-6/*n*-3 and PUFA/SFA has been suggested as useful in evaluations regarding the nutritional quality of fish lipids [19,54,59]. Values of the PUFA/SFA and *n*-6/*n*-3 ratios above 0.45 and below 4.0, respectively, have been recommended by the UK Department of Health and Social Security [60] and the UK Department of Health [61]. The maintenance of these rates is caused by permanent and balanced fish lipid ingestion and leads to the prevention of cardiovascular disorders. The values of PUFA/SFA (2.11–3.47) and *n*-6/*n*-3 (0.66–1.28) ratios obtained in the present study indicated benefits in the consumption of all fish species [20]. Similar PUFA/SFA results were verified for *Esox lucius* (2.46) [59] and *Lates niloticus* (3.15) [62], whereas similar *n-*6/*n-*3 values were observed for *Prochilodus* spp. (1.03) [8] and *Cichla* sp. (1.02–1.50) [11] as well as for different freshwater fish species (0.21–1.0) [54]. Regarding the *n*-3/*n*-6 ratio all species were considered well balanced for human nutrition when compared to the range of 1:1–1:5 suggested by Memon et al. [19]. Moreover, the same authors suggested that high *n*-3/*n*-6 ratios are involved in health benefits. However, CO (1.52) presented the highest (*p* < 0.05) value and was considered the major relevant species in this regard. Accordingly, Mert et al. [59] also observed high *n*-3/*n*-6 ratios in *E*. *lucius* during different seasons (1.33–1.97), as did Memon et al. [19] in *L*. *rohita*, *C*. *mrigala* and *C*. *catla* (1.69–1.91).

The indices of atherogenicity (IA) and thrombogenicity (IT) are involved in platelet aggregation potential and subsequent formation of thrombus and atheroma in the cardiovascular system [53]. Thus, lower IA and IT values are desirable to prevent cardiovascular disorders. Similar IA (0.19–0.34) reports and higher IT (0.06–0.11) values were observed by Volpe et al. [53] in *O*. *mykiss* (IA = 0.16; IT: 0.18). The lower IT values found in the present study could be related to the higher PUFA amounts present in the studied species (50.91−58.70) in comparison to PUFA values reported for *O*. *mykiss* (44.26) [53]. The h/H ratio indicates the proportion of ∑ hypocholesterolemic/hypercholesterolemic FA and its relation to the cholesterol metabolism [20]. Thus, in contrast to IA and IT, high h/H values are recommended to provide benefits for human health. Among fish species studied, BC presented the greatest (*p* < 0.05) h/H values, exceeding values reported for marine fish (0.87–2.46) [20]. The NQI results of the present study highlight the relevance of the consumption of this fish species regarding the prevention of several vascular disorders.

PCA test was performed in order to detect any natural clustering among fish species and indicate most desirable group for human consumption. According to PCA results, it was possible to verify a relationship between BC and CO, due to the presence of more expressive content of healthy NQI indicators. This similarity was not related to proximity of trophic guilds but to specific metabolisms, as well as the anatomic and physiological features of each genus. The Brycon (BC) genus exhibits metabolic adaptations of intestinal enzymes that allows for physiological adjustments and optimal use of food nutrients [40], whereas the Cichla (CO) genus presents intestinal characteristics that assist in higher intestinal absorption, in addition to the maintenance of a great amount of enzymatic apparatuses for EPA and DHA synthesis and DHA accumulation [41,55]. These results classify the aforementioned species as the most desirable for human consumption and corroborate the NQI and fatty acid profile findings of this study. Nevertheless, CO obtained the best performance among the evaluated species, since they are more close to *n*-3 PUFA and its associated indices (DHA/EPA, EPA+DHA and *n*-3/*n*-6), which are strongly related to the prevention of cardiovascular diseases and increased health benefits.

In conclusion, a significant relationship and similar lipid nutritional value were verified in two fish species of the families investigated herein, represented by *Brycon cephalus* and *Cichla ocellaris*. These species presented high PUFA content, especially *n*-3 FA, while DHA accounted more than 35% of total PUFA content. In addition, optimum NQI values were observed, characterizing these as relevant quality matrices, desirable to human health. Nonetheless, the nutritional quality indices of all the studied species were within the recommended standard, indicating that their consumption can provide several benefits for human health, mainly related to the decrease of cardiovascular disease risks. Thus, greater incentives regarding the consumption and exploitation of these species should be encouraged, since they could augment the development of freshwater aquaculture, as well as increase fish intake, improving human diet balances and, consequently, human nutrition.

## Abbreviations

FA: fatty acids
BC: Brycon cephalus
CO: Cichla ocellaris
PL: Prochilodus lineatus
LF: Leporinus friderici
PCO: Pseudoplatystoma corruscans
SFA: saturated fatty acids
MUFA: monounsaturated fatty acids
PUFA: polyunsaturated fatty acids
EPA: eicosapentaenoic acid
DHA: docosahexaenoic acids
*n*-3: omega-3 fatty acid
*n*-6: omega-3 fatty acid
LA: linoleic acid
FAME: fatty acids methyl esters
IA: index of atherogenicity
IT: index of thrombogenicity
h/H: hypocholesterolemic/hypercholesterolemic ratio
PCA: principal component analysis
PC1: principal component-1
PC2: principal component-2
NQI: nutritional quality indices
